# Emerging Targets in Psoriatic Arthritis: Dual IL-17A/F and Selective TYK2 Inhibition in a Clinical Perspective

**DOI:** 10.31138/mjr.101025.erh

**Published:** 2026-01-08

**Authors:** Violeta Dimopoulou, Maria Nitsa, Christos Koutsianas

**Affiliations:** Clinical Immunology – Rheumatology Unit, 2^nd^ Department of Medicine and Laboratory, National and Kapodistrian University of Athens, School of Medicine, “Hippokration” General Hospital of Athens, Greece

**Keywords:** bimekizumab, deucravacitinib, interleukin 17A/F, psoriatic arthritis, targeted therapy, TYK2

## Abstract

Psoriatic arthritis (PsA) is a multifaceted immune-mediated inflammatory disease with several unmet therapeutic needs, and many patients fail to achieve comprehensive disease control with existing treatments. This has driven the exploration of novel therapeutic targets deriving from a better understanding of the immunopathogenetic cascade. Two of the most promising emerging targets are the dual inhibition of interleukin-17A and F (IL-17A/F) and the selective inhibition of tyrosine kinase 2 (TYK2). Bimekizumab, a monoclonal antibody that neutralises both IL-17A and IL-17F, has demonstrated superior efficacy over placebo and a standard-of-care TNF inhibitor in pivotal Phase III trials, achieving high rates of both joint and skin response. Its safety profile is characterised by a manageable increase in mild-to-moderate fungal infections, particularly oral candidiasis. Deucravacitinib, an oral, allosteric TYK2 inhibitor, represents a novel mechanism of action that modulates key cytokine pathways (IL-23, IL-12, Type I IFN) without inhibiting JAKs 1-3. Phase II and preliminary phase III data in PsA show significant improvements in joint and skin symptoms, with a safety profile from long-term psoriasis studies that appears favourable, showing minimal hematologic or laboratory abnormalities. The advent of bimekizumab and deucravacitinib enriches the PsA treatment arsenal and their introduction will help with more personalised management strategies for patients with PsA.

## INTRODUCTION

Psoriatic arthritis (PsA) is a chronic, immune-mediated inflammatory disease that manifests across a spectrum of domains, including peripheral and axial joints, skin, nails, and entheses. With a prevalence of up to 30% in patients with psoriasis, PsA can lead to progressive joint damage, functional disability, and a substantial impairment in quality of life.^1,2^ The pharmacological management of PsA has evolved considerably over the past two decades, moving from conventional synthetic disease-modifying antirheumatic drugs (csDMARDs) to biologic (bDMARD) and targeted synthetic (tsDMARD) agents targeting specific cytokine pathways, such as tumour necrosis factor-alpha (TNF-α), interleukin (IL)-17A, and the IL-23/IL-17 axis, as well as intracellular kinases via Janus kinase (JAK) inhibition.^3^ Despite these advances, a significant proportion of patients experience an inadequate response, loss of efficacy over time, or intolerance to existing therapies.^4^

This persistent unmet need has fuelled research into more precise interventions that target critical nodes in the PsA pathophysiology. Two such targets have recently come to the fore: the dual inhibition of the closely related cytokines IL-17A and IL-17F^5^ and the selective inhibition of tyrosine kinase 2 (TYK2).^6^ It has been several years since the role of the IL-17 family of cytokines has been recognised as central to the pathogenesis of PsA. There are recent evidence underscoring the synergistic role of IL-17F in driving chronic inflammation and tissue pathology.^7,8^ Parallel to this, the recognition of TYK2’s crucial role as an intracellular mediator for key cytokines upstream of IL-17, including IL-23 and Type I interferons, offers a distinct yet complementary therapeutic strategy.^9^

This clinical review aims to collate the current evidence for two agents targeting these pathways: bimekizumab, a humanised monoclonal antibody inhibiting both IL-17A and IL-17F, and deucravacitinib, an oral, selective allosteric TYK2 inhibitor. This review provides a contemporary comparative synthesis of these two emerging mechanistic classes offering clinical insights into their positioning within the evolving PsA treatment paradigm.

The authors performed a search on PubMed/Medline, Scopus, and Embase using the following terms: [(“Psoriatic Arthritis” OR “psoriasis”) AND (“Bimekizumab” OR “IL-17A” OR “IL-17F” OR “Interleukin-17A” OR “Interleukin-17” OR “dual inhibition” OR “anti-IL-17”) OR (“Deucravacitinib” OR “BMS-986165” OR “TYK2” OR “tyrosine kinase 2”)]. The search results were critically appraised to inform a narrative review on the mechanistic rationale, clinical efficacy across PsA domains, and safety profiles of bimekizumab and deucravacitinib, concluding with a discussion on their significance in future treatment paradigms for PsA patients.

## BIMEKIZUMAB AND THE RATIONALE FOR DUAL IL-17 A AND F INHIBITION

The interleukin-17 family consists of six members (IL-17A through IL-17F). IL-17A and IL-17F form homodimers or heterodimers (IL-17A/F) that signal through the same IL-17RA/RC receptor complex.^10^ Historically, therapeutic blockade has focused on IL-17A alone. However, a growing body of preclinical and clinical data suggests that IL-17F is an active and synergistic cytokine, contributing to psoriatic disease inflammation.^11^ IL-17A is a potent inducer of pro-inflammatory mediators from stromal and immune cells, leading to neutrophil recruitment, osteoclastogenesis, and the production of antimicrobial peptides and chemokines that perpetuate inflammation in the joint and skin. IL-17F, while less potent than IL-17A on a molecular basis, is often expressed at higher levels in psoriatic plaques and synovial tissue and demonstrates overlapping functions.^12^ Preclinical data demonstrated that blocking IL-17A alone reduced the production of pro-inflammatory mediators, such as IL-8, in stimulated synoviocytes from PsA patients, while blocking IL-17F alone had no significant impact. However, dual inhibition of IL-17A and IL-17F resulted in greater suppression of pro-inflammatory cytokines: IL-6 (42% reduction, <0.01), IL-8 (28% reduction, p<0.05), and MMP3 (44% reduction, p=0.149) compared to IL-17A blockade alone.^12^ This suggests that IL-17F may contribute independently to the inflammatory load and that co-inhibition could provide a more comprehensive suppression of the IL-17 pathway.

### Mechanism of Action

Bimekizumab is a humanised immunoglobulin G1/κ (IgG1/κ) monoclonal antibody, whose unique structure features two identical antigen-binding fragments (Fabs). This allows a single bimekizumab molecule to simultaneously neutralize IL-17A and IL-17F homodimers as well as the IL-17A/F heterodimer, effectively preventing all of their interaction with the IL-17RA/RC receptor complex and subsequent downstream signalling. This direct, dual neutralisation is the defining characteristic of its mechanism of action. Bimekizumab exhibits linear and dose-proportional pharmacokinetics, with an average half-life of approximately 24 days.^12^ Bimekizumab is capable of binding with high affinity to both IL-17A and IL-17F, regardless of their respective concentrations.^13^

### Clinical efficacy

The clinical development program of bimekizumab in PsA has been comprehensive, progressing from proof-of-concept to large, pivotal Phase III trials.

#### Phase I and II Trials

The initial proof-of-concept study (NCT02141763) demonstrated a rapid and profound efficacy signal starting as early as week 2 of treatment. At Week 8, patients receiving the higher doses of bimekizumab achieved an ACR20/ACR50/ACR70 response rate of 80/40/23.3 % compared to 16.7/8.3/0 % for placebo.^12^ Notably, complete skin clearance (PASI100) was achieved in 87% of patients with significant skin involvement, underscoring bimekizumab’s potent effect on skin involvement.

The subsequent Phase IIb, dose-ranging BE ACTIVE trial (NCT02969525)^14^ established the 160 mg every 4 weeks (with or without a loading dose) as the optimal dosing regimen. The primary endpoint of ACR50 at Week 12 was achieved by 41 and 46% of patients in the key bimekizumab groups compared to 7% for placebo (p<0.001). Efficacy was sustained through the 48-week blinded period and the open-label extension (OLE), with over half of the patients achieving ACR50 and Minimal Disease Activity (MDA) at Week 152, demonstrating remarkable long-term durability.^15^

#### Phase III Trials

**Figure 1.**
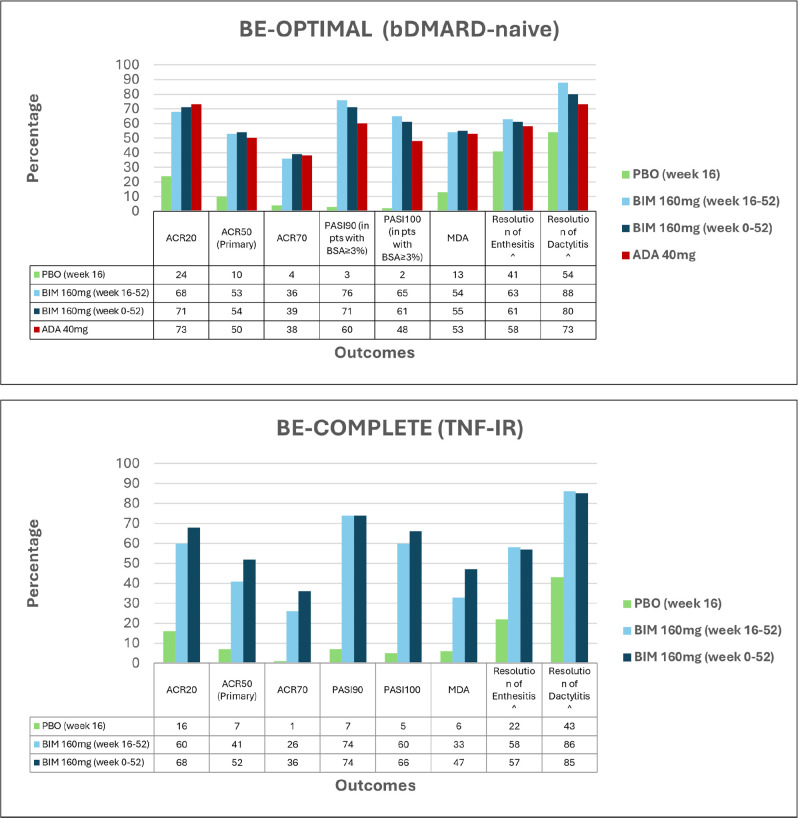
Key efficacy outcomes of bimekizumab in Phase III PsA trials at week 52. BIM: Bimekizumab; PBO: Placebo; ADA: Adalimumab; TNFi-IR: TNF Inhibitor Inadequate Responder; *p<0.0001 vs. placebo. ^ Among patients with condition at baseline.

The preliminary phases’ data for bimekizumab was confirmed and expanded in two pivotal studies examining specific populations: bDMARD-naïve patients (BE OPTIMAL, NCT03895203) and those with inadequate response to TNF inhibitors (BE COMPLETE, NCT03896581) (**Figure 1**). In BE OPTIMAL,^16^ which included an adalimumab reference arm, 44% of bimekizumab-treated patients achieved ACR50 at Week 16, compared to 10% for placebo (OR 7.1; p<0.0001) and 46% for adalimumab. The skin response proved again to be striking: 65% of bimekizumab patients with ≥3% BSA involvement achieved PASI100 versus 11% with placebo (p<0.0001). In BE COMPLETE,^17^ which enrolled TNF-inhibitor inadequate responders, 43% of bimekizumab-treated patients achieved ACR50 at Week 16 compared to 7% on placebo (OR 11.1; p<0.0001). This robust response in TNF-inhibitor inadequate responders highlights its effectiveness after prior biologic exposure.

Long-term efficacy has been consistently maintained across both studies and their OLEs with data reaching up to 3 years. In bDMARD-naïve patients, ACR50 responses were sustained at 55% at week 52^18^ and at 53.2% at week 160.^19^ Similarly, among TNFi-experienced PsA patients who started bimekizumab at baseline, 52% ACR50 at week 52^20^ and 55% ACR50 at week 156.^21^ High rates of PASI100 (62–67%) and MDA (44–53%) response were also preserved up to the third year of treatment with bimekizumab.^18–21^ Furthermore, bimekizumab has data to support its disease modifying role, since it has demonstrated significant inhibition of radiographic progression in the BE OPTIMAL study, with 87–89% of patients receiving BKZ showing no progression (change in van der Heijde-modified Total Sharp Score [vdH-mTSS] ≤0.5) at week 52,^18^ extending to week 104 with a percentage of 79–84%.^22^ Importantly, these clinical improvements translated into significant and clinically meaningful benefits in patient-reported outcomes (PROs), including pain, fatigue, physical function (HAQ-DI), and health-related quality of life (SF-36) through 52 weeks of treatment.^20^ Finally, these clinical improvements translated into significant and clinically meaningful benefits in patient-reported outcomes (PROs), including pain, fatigue, physical function (HAQDI), and health-related quality of life (SF-36) as well as work productivity through 52 weeks of treatment,^23^ in both bDMARD-naïve and TNFi-experienced patients.

### Safety and tolerability of bimekizumab

The safety profile of bimekizumab has been well characterised across its clinical trial program, with consistent findings from Phase I through to Phase III studies and their long-term extensions (**Table 1**). In the pooled analysis of the Phase III trials (BE OPTIMAL and BE COMPLETE) up to Week 104, bimekizumab demonstrated a generally manageable safety profile.^16,17,24,25^

**Table 1.** Key safety outcomes of bimekizumab in Phase III PsA trials (pooled analysis, week 0–52).

	**BE OPTIMAL (bDMARD-naïve)**		**BE-COMPLETE (TNF-IR)**
**Safety Event**	**BIM 160 mg Q4W (n =** 702**)** EAIR (95%CI) / 100 PY	**ADA 40 mg Q2W (n = 140)** EAIR (95%CI) / 100 PY	**BIM 160 mg Q4W (n =** 702**)** EAIR (95%CI) / 100 PY
**Any TEAE**	223.0 (204.8, 242.3)	209.4 (172.6, 251.7)	125.2 (110.0, 142.0)
**Serious TEAEs**	7.8 (5.7, 10.4)	7.5 (3.6, 13.9)	7.0 (4.4, 10.4)
**Discontinuation due to AE**	4.1 (2.6, 6.1)	5.2 (2.1, 10.8)	5.1 (3.0, 8.1)
**Deaths**	0.2 (0.0, 0.9)	0	0.3 (0.0, 1.6)
**Serious Infections**	1.0 (0.4, 2.2)	1.5 (0.2, 5.3)	2.1 (0.8, 4.3)
**Any Fungal Infection**	15.3 (12.2, 18.9)	1.5 (0.2, 5.3)	12.2 (8.7, 16.7)
**• *Oral Candidiasis***	6.7 (4.8, 9.2)	0.7 (0.0, 4.1)	7.6 (4.9, 11.3)
**IBD Events**	0.7 (0.2, 1.7)	0	0
**MACE**	0.7 (0.2, 1.7)	0	0.9 (0.2, 2.6)
**Incidence of Malignancy (excluding NMSC)**	0.7 (0.2, 1.7)	0	0
**Uveitis**	0	0	0

BIM: Bimekizumab; ADA: Adalimumab; TNFi-IR: TNF Inhibitor Inadequate Responder; EAIR: Exposure-Adjusted Incidence Rate; PY: Patient-Years; TEAE: Treatment-Emergent Adverse Event; MACE: Major Adverse Cardiovascular Event; IBD: Inflammatory Bowel Disease; NMSC: Non-Melanoma Skin Cancer. Both deaths were judged unrelated to study treatment by investigators.

Treatment-emergent adverse events (TEAEs) were common, largely non-serious and remained consistent from weeks 0–52 to weeks 52–104. The most frequently reported TEAEs with bimekizumab were nasopharyngitis and upper respiratory tract infections, including SARS-Cov-2. The most notable finding is an increased incidence of generally mild-to-moderate fungal infections, particularly oropharyngeal candidiasis, which is a predictable consequence of bimekizumab’s potent dual inhibition of the IL-17 pathway, a key mediator immunity against fungi. The exposure-adjusted incidence rate (EAIR) of any fungal infection across the Phase III program was approximately 10–12 per 100 patient-years (PY). The vast majority of these were Candida infections (EAIR ~7–8 per 100 PY), with oral candidiasis being the most common manifestation. These events were typically mild or moderate in severity, localised, and resolved with standard antifungal therapy; there were no systemic Candida infections reported. Very few fungal infections (<1%) led to discontinuation of treatment and no cases of drug discontinuation due to recurrent fungal infections were noted.

Serious infections were reported at a low frequency (EAIR ~1–2 per 100 PY), while there was no signal with regards to opportunistic infections. The incidence of inflammatory bowel disease (IBD) and major adverse cardiovascular events (MACE) was low and consistent with the background rates expected in a PsA population. The incidence rate of malignancies (excluding non-melanoma skin cancer) in the bimekizumab studies was also low (EAIR ~ 0.4–0.9 per 100 PY). No cases of uveitis were noted during the program. Overall, there were no new safety signals that emerged after long-term treatment for up to 3 years.^20,24,26^

### Deucravacitinib and the rational for selective TYK2 inhibition

For decades cytokine blockade at the receptor level has been a cornerstone of the treatment of rheumatic diseases, including PsA. Targeting intracellular signalling pathways offers a distinct therapeutic approach and the ability to block the effect of several cytokines with one agent. Tyrosine kinase 2 (TYK2) is a member of the Janus kinase (JAK) family and serves as a crucial signalling mediator for several cytokines implicated in PsA pathogenesis, including interleukin-23 (IL-23), IL-12, and Type I interferons (IFNs).^27^ These cytokines are pivotal in the differentiation and maintenance of T-helper (Th) 17 and Th1 cells, which are key drivers of inflammation in psoriatic disease.^28^ The IL-23/IL-17 axis is of particular importance. IL-23, signalling through a receptor complex utilizing TYK2 and JAK2, promotes the expansion of Th17 cells, which subsequently produce IL-17A, IL-17F, and other pro-inflammatory mediators. This leads to proliferation and activation of synovial fibroblasts and keratinocytes, creating a self-perpetuating inflammatory loop leading to chronic joint inflammation and, finally, joint destruction. Therefore, inhibiting TYK2 modulates this pathway upstream of IL-17 production.^29,30^

### Mechanism of Action

Traditional JAK inhibitors are orthosteric and target the conserved ATP-binding catalytic site of JAK enzymes, often inhibiting multiple JAK family members (JAK1, JAK2, JAK3), which can lead to a broad range of off-target effects, such as haematological abnormalities and increased risk of infections, while there is still controversy about their effect on cardiovascular risk.^31^

Deucravacitinib represents a paradigm shift through its unique mechanism of action. It is a first-in-class, oral, selective TYK2 inhibitor that binds with high specificity to the regulatory (pseudokinase) domain JH-2 of TYK2, allosterically stabilizing an inhibitory conformation.^32,33^ This highly selective binding inhibits TYK2-dependent cytokine signalling without appreciably inhibiting JAK1, JAK2, or JAK3, thereby minimising the risks associated with broader JAK inhibition and offering a potentially optimised benefit-risk profile.^34^

### Clinical efficacy

The efficacy of deucravacitinib in psoriasis has already been established in randomized, double-blind, placebo-controlled trials^35,36^ and their extensions,^37^ showing PASI75 responses superior to other conventional synthetic DMARDs (such as methotrexate and apremilast) and comparable to TNF inhibitors (such as etanercept and infliximab).^38^

Deucravacitinib data focusing in active PsA was provided by a Phase II, randomised, double-blind, placebo-controlled trial (NCT03881059).^39^ Patients with active PsA (≥3 tender and ≥3 swollen joints) and an inadequate response to prior therapies (including csDMARDs and/or one TNF inhibitor) were randomised to receive placebo, deucravacitinib 6 mg, or deucravacitinib 12 mg once daily for 16 weeks. The primary endpoint of the ACR-20 response was significantly higher in the 6 mg (52.9%) and 12 mg (62.7%) groups compared to placebo. Significant improvements were also observed in key secondary endpoints, including ACR50, PASI75 (in patients with ≥3% body surface area involvement), and physical function as measured by HAQ-DI (**Table 2**). Deucravacitinib’s efficacy was not influenced by baseline csDMARD use and monotherapy patients had similar ACR20 response rates when compared to combination treatment.^40^ A post-hoc analysis of this trial further demonstrated that a greater proportion of patients treated with deucravacitinib achieved Minimal Disease Activity (MDA) and met the criteria for individual MDA components, with a particularly strong effect on tender joint count, compared to placebo.^41^

**Table 2.** Efficacy of deucravacitinib in a Phase II trial in active PsA (week 16).

**Endpoint**	**Placebo (n=66)**	**Deucravacitinib 6 mg (n=70)**	**Deucravacitinib 12 mg (n=67)**
**ACR20 (Primary)**	31.8%	52.9%*	62.7%**
**ACR50**	10.6%	24.3%*	32.8%**
**ACR70**	1.5%	14.3%	19.4%*
**PASI75 (in pts with BSA≥3%)**	20.4%	42.4%**	59.6%**
**HAQ-DI MCID (≥0.35)**	15.2%	38.6%*	40.3%**
**MDA**	7.6%	22.9%*	23.9%*
**Resolution of Enthesitis (LEI=0)^**	22.6%	51.3%	50.0%*
**Resolution of Dactylitis (LDI=0)^**	60.0%	76.7%	79.2%*

pts: patients; MCID: Minimal Clinically Important Difference.

** p<0.05,

* p<0.01 vs. placebo.

^ Among patients with condition at baseline

Based on the data above, deucravacitinib appears as a promising treatment for PsA and it is already being further investigated in larger and longer-term phase III trials (POETYK-PsA1 and POETYK-PsA2). The preliminary data up to week 16 from POETYK-PsA1 appears to be confirming the efficacy seen in the phase II trial, with ACR20 response at 54.2% for the 6mg dose versus 34.1% for placebo (p<0.0001) and similar responses for secondary endpoints, such as ACR50, ACR70, PASI75 and patient-reported outcomes.^42^

### Safety

The unique mechanism of action of deucravacitinib and its high selectivity for TYK2 should, in theory, reduce off-target effects and thus provide the drug with a favourable adverse event profile.^43^ This mechanistic assumption appears to be confirmed by clinical data both in plaque psoriasis and PsA.

Long-term safety data is available from the extensive POETYK clinical program in plaque psoriasis, where deucravacitinib has been evaluated for up to 4 years.^37^ In this population, the safety profile remained consistent over time. The EAIRs for serious AEs were 5.0 per 100 PY over 4 years. The incidence rates for AEs of special interest, including serious infections, herpes zoster, MACE, VTE, and malignancies, were low and comparable to rates observed with placebo in the controlled periods and with non-biologic systemic therapies.^37,44^ In the 16-week Phase II PsA trial, deucravacitinib demonstrated a favourable safety profile PsA.^39,42^ The most common AEs were nasopharyngitis, upper respiratory tract infection, headache, and diarrhoea, which were generally mild to moderate in severity. Once again, there were no serious infections, herpes zoster reactivations, opportunistic infections, MACEs or VTEs reported in the deucravacitinib groups.

This accumulating long-term data provides reassuring support for the continued use of deucravacitinib in clinical practice.

### Therapeutic positioning and future perspectives

The development of bimekizumab and deucravacitinib represents significant advancement in the therapeutic arsenal for PsA, each targeting distinct yet interconnected nodes in the disease’s immunopathogenesis and providing different advantages. Their respective targets are validated, and this compels a re-evaluation of treatment sequences and personalised management strategies.

#### Efficacy Across Domains and Populations

Bimekizumab has established a new benchmark for efficacy, particularly in patients with concurrent significant skin involvement. The rates of complete skin clearance (PASI100) observed in the Phase III trials— approximately 65% in bDMARD-naïve patients and over 50% in TNF-inhibitor inadequate responders—are among the highest reported for any biologic therapy in PsA to date. This, coupled with robust joint responses (ACR50 >40%), inhibition of radiographic progression, and meaningful improvements in patient-reported outcomes, positions it as a leading choice for patients where skin disease is a dominant concern. Its efficacy in resolving enthesitis and dactylitis further underscores its comprehensive domain coverage.^45^ Recent network meta-analyses of RCTs have shown that bimekizumab was associated with the highest surface under the cumulative ranking curve (SUCRA) for efficacy outcomes among other advanced therapies.^46,47^

Deucravacitinib, while its data is evolving, has demonstrated a very encouraging efficacy signal from its Phase II and preliminary phase III trials. The ACR20 and PASI75 response rates place it firmly in the realm of advanced therapies. Its oral route of administration is a significant advantage for patient preference and convenience. In a network meta-analysis comparing different advanced therapies in plaque psoriasis, deucravacitinib exhibited lower SUCRA for PASI 90 compared to other biologic classes, but better compared to apremilast.^48^

#### Safety and Tolerability

The safety profiles of these agents are a direct reflection of their mechanisms of action. For bimekizumab, the increased incidence of oropharyngeal candidiasis is a class-effect of the profound IL-17 pathway inhibition, given the crucial role of this cytokine in mucosal antifungal defense.^49,50^ However, these events are manageable in clinical practice, rarely leading to discontinuation, and can often be anticipated and addressed with patient education and prompt antifungal treatment. The low incidence of other AEs of interest, such as IBD and uveitis exacerbation and MACE, is reassuring.

For deucravacitinib, the emerging safety profile appears distinct from that of broader JAK inhibitors. The absence of significant hematologic abnormalities, such as neutropenia or anaemia, in clinical trials to date is consistent with its lack of inhibition of JAK2 (critical for erythropoietin signalling) and JAK3 (involved in lymphocyte development)^51^. Similarly, the low rates of herpes zoster may relate to its sparing of JAK1-dependent IFN signalling, which is important for viral immunity. This differentiated profile is a key part of its value proposition.

#### Positioning in the Treatment Landscape: A Clinical Perspective

The introduction of these agents provides more therapeutic choices for clinicians managing patients with PsA.

Bimekizumab is poised for use in patients requiring the highest level of efficacy, especially those with severe skin psoriasis alongside active PsA. It is a compelling option both as a first-line biologic and after failure of other mechanism classes, such as TNF inhibitors. Its potent and rapid skin clearance can be a decisive factor. The clinical results on efficacy and safety have led to characterising dual IL17A/F inhibition as a mechanism of action distinct to the up until now available IL17A inhibitors.^3,52^

Deucravacitinib’s positioning is more complex and will be clarified by the full Phase III trial data. Based on current evidence, we propose two primary contexts: Firstly, as a superior oral alternative to apremilast; for patients who have failed csDMARDs and prefer an oral therapy, deucravacitinib offers a mechanistically distinct and more effective option than apremilast with a different side effect profile (e.g., less gastrointestinal disturbance, but potentially more upper respiratory infections). Secondly, as a selective oral agent versus broader JAK inhibitors; within the class of oral targeted synthetic DMARDs, deucravacitinib’s TYK2 selectivity may offer a safety advantage for patients concerned about the hematologic and potential cardiovascular risks associated with JAK1-3 inhibition. It could be particularly attractive for patients with contraindications or apprehensions about broader JAK inhibition, even though longer data is required for further clarification of this notion.

The choice between an injectable like bimekizumab and an oral therapy like deucravacitinib will also hinge on patient preference, comorbidities, and cost/access considerations. The potential for combination therapy, for instance using deucravacitinib with a biologic, remains an unexplored but intriguing area for future research, potentially offering synergistic efficacy for refractory disease.

## CONCLUSION

The landscape of PsA treatment is being reshaped by therapies that offer increasingly precise and potent inhibition of key pathogenic pathways. Bimekizumab, through its dual neutralisation of IL-17A and IL-17F, delivers profound efficacy across musculoskeletal and cutaneous domains, setting a new standard for comprehensive disease control, albeit with a manageable and mechanism-based risk of candidiasis. Deucravacitinib, as a first-in-class, allosteric TYK2 inhibitor, provides a novel oral option that effectively modulates the IL-23/IL-17 axis upstream, with a promising efficacy signal and a potentially differentiated safety profile from broader JAK inhibitors.

Together, these agents significantly address some of the unmet needs in PsA. They provide clinicians with powerful new tools to achieve treatment goals, allowing for more tailored therapeutic strategies that can be aligned with individual patient disease profiles, preferences, and risk tolerances.
